# Identification of *Streptococcus cristatus* peptides that repress expression of virulence genes in *Porphyromonas gingivalis*

**DOI:** 10.1038/s41598-017-01551-4

**Published:** 2017-05-03

**Authors:** Meng-Hsuan Ho, Richard J. Lamont, Hua Xie

**Affiliations:** 1 Department of Oral Biology, Meharry Medical College, Nashville, TN 37208 United States; 2 Department of Oral Immunology and Infectious Diseases, University of Louisville, Louisville, KY 40202 United States

## Abstract

Dental plaque is a complex multispecies biofilm, and is a direct precursor of periodontal disease. The virulence of periodontal pathogens, such as *Porphyromonas gingivalis*, is expressed in the context of this polymicrobial community. Previously, we reported an antagonistic relationship between *Streptococcus cristatus* and *P*. *gingivalis*, and identified arginine deiminase (ArcA) of *S*. *cristatus* as the signaling molecule to which *P*. *gingivalis* responds by repressing the expression and production of FimA protein. Here we demonstrate that direct interaction between *P*. *gingivalis* and *S*. *cristatus* is necessary for the cell-cell communication. Two surface proteins of *P*. *gingivalis*, PGN_0294 and PGN_0806, were found to interact with *S*. *cristatus* ArcA. Using a peptide array analysis, we identified several *P*. *gingivalis*-binding sites of ArcA, which led to the discovery of an 11-mer peptide with the native sequence of ArcA that repressed expression of fimbriae and of gingipains. These data indicate that a functional motif of ArcA is sufficient to selectively alter virulence gene expression in *P*. *gingivalis*, and PGN_0294 and PGN_0806 may serve as receptors for ArcA. Our findings provide a molecular basis for future rational design of agents that interfere with the initiation and formation of a *P*. *gingivalis*-induced pathogenic community.

## Introduction

Periodontitis is the 6^th^ most common infection worldwide^1^ and an estimated 5–20% of the population suffers from generalized chronic periodontitis^2, 3^. In the US, around half of the adult population suffers from some form of periodontal disease, which constitutes a significant economic burden^4^. Periodontal diseases and periodontal bacteria are also physically and epidemiologically associated with severe systemic conditions such as coronary artery disease, rheumatoid arthritis, and diabetes^5–7^. Chronic periodontitis is the result of a breakdown of periodontal tissue-microbe homeostasis, which then leads to uncontrolled inflammation and tissue destruction^8^. Loss of tissue homeostasis, called dysbiosis, is initiated by communities of organisms colonizing the subgingival area. In most instances these microbial communities are associated with health, and the transition to pathogenesis requires colonization by specific pathogens such as *Porphyromonas gingivalis*. It has long been recognized that the presence of *P*. *gingivalis* alone is insufficient for the disease to occur in a susceptible host, and current models hold that *P*. *gingivalis* is a keystone pathogen in that it elevates the virulence of the entire community^9–12^. Evidence for this comes from murine models in which low levels of *P*. *gingivalis* can initiate disease, but only in the context of a microbial community^9^, and from primate studies where a gingipain-based vaccine caused a reduction both in *P*. *gingivalis* numbers and in the total subgingival bacterial load, as well as in inhibiting bone loss^13^.

Bacteria within the oral microbial community can exhibit polymicrobial synergy whereby interspecies communication enhances colonization and pathogenic potential. Conversely, oral organisms also engage in antagonistic interactions, whereby one organism inhibits the colonization or growth of another. For example, communities of *P*. *gingivalis* and *Streptococcus gordonii* are synergistically pathogenic in a murine model of alveolar bone loss^14^, whereas *Streptococcus cristatus* interferes with the colonization and pathogenesis of *P*. *gingivalis* in mice^15^. We, and others, have reported previously that arginine deiminase (ArcA) of *S*. *cristatus* represses expression of the major fimbrial adhesin of *P*. *gingivalis*, FimA^16, 17^. ArcA catalyzes the hydrolysis of L-arginine to L-citrulline and ammonia, and the latter is believed to be important for oral biofilm pH homeostasis and the prevention of caries^18, 19^. We also found that the expression of *arcA* was significantly higher in *S*. *cristatus* than in *S*. *gordonii* ^20^, suggesting that ArcA activity could define the differing roles of these two streptococcal species in the highly orchestrated formation of dental plaque. Moreover, clinical studies revealed an inverse relationship between the numbers of *S*. *cristatus* compared to *P*. *gingivalis* cells in dental plaque isolated from periodontitis subjects, suggesting that *S*. *cristatus* may be beneficial to the host by antagonizing the colonization and accumulation of *P*. *gingivalis* ^21^.

In this study, we investigated the components of the antagonistic communication system between *P*. *gingivalis* and *S*. *cristatus* including functional motifs of ArcA and receptor(s) of *P*. *gingivalis*. We found that direct contact is required in intergeneric communication between *P*. *gingivalis* and *S*. *cristatus*. Our results also identified a short linear domain of ArcA as the binding site for *P*. *gingivalis*. Two surface proteins of *P*. *gingivalis*, PGN_0294 (RagB) and PGN_0806, likely serve as receptors in this bacterial cell-cell communication. Equally important, we were able to show that the short ArcA-derived peptide represses expression of several established virulence genes of *P*. *gingivalis* including *fimA*, *mfa1*, *rgpA*, *rgpB*, and *kgp*. Exploitation of this antagonistic relationship may lead to the discovery of pharmaceutical agents to inhibit *P*. *gingivalis* colonization and pathogenicity.

## Results

### Direct contact is required for *P*. *gingivalis*-*S*. *cristatus* communication

**Figure 1:**
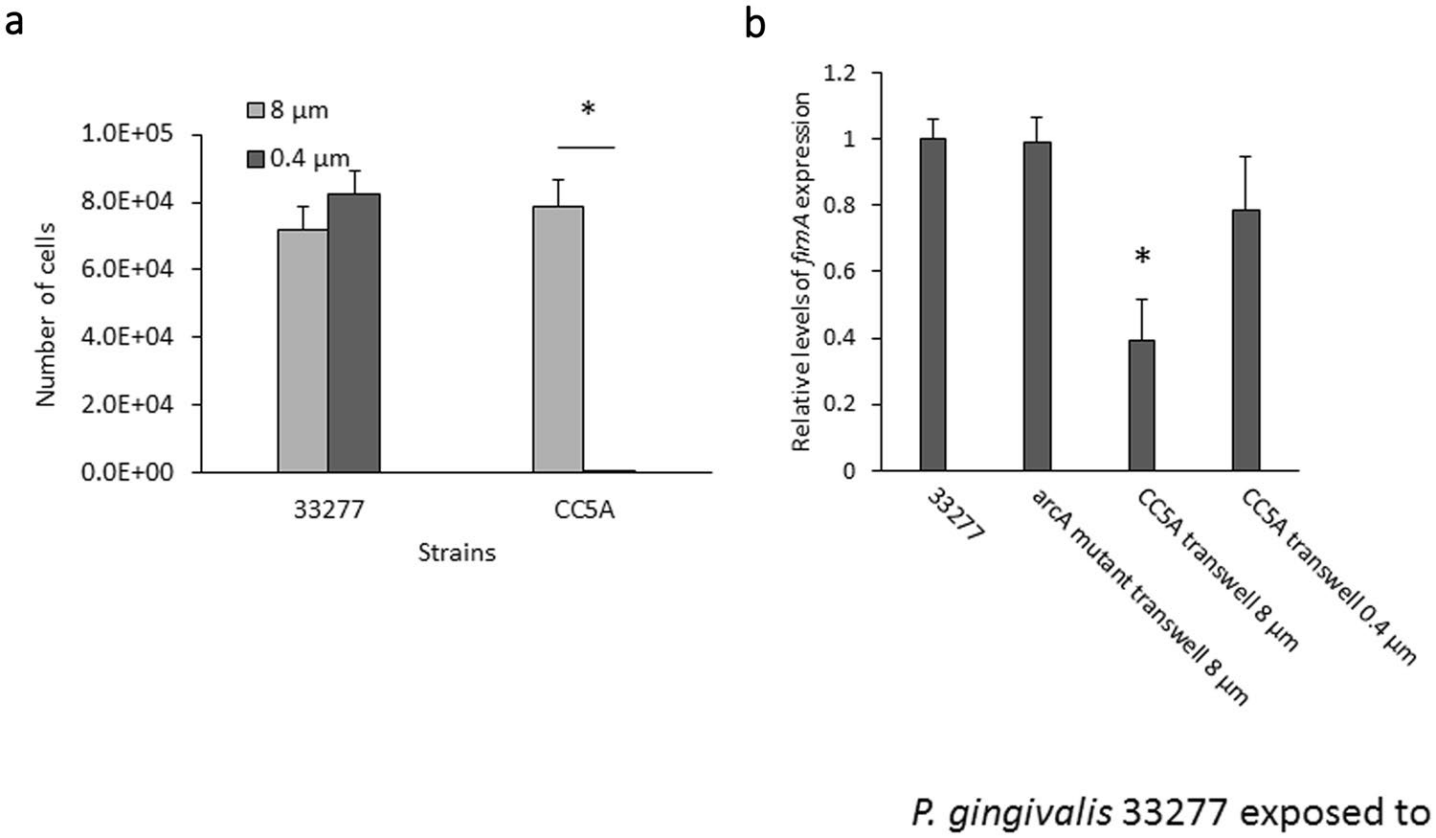
Expression of *fimA* in *P*. *gingivalis* in contact with *S*. *cristatus*. (**a**) *S*. *cristatus* CC5A migration through the Transwell insert. CC5A (1 × 10^7^ cells) were initially incubated in the Transwell insert, and the numbers of CC5A migrated to the lower well and 33277 cells in the well were determined by qPCR. Each bar represents the number of bacteria detected in the lower well. Asterisk indicates a statistically significant difference in number of bacteria in the lower wells (n = 3; *t* test; *p* < 0.05). (**b**) Expression of the *fimA* gene in *P*. *gingivalis* in transwell chambers with *S*. *cristatus* was measured by real-time qRT-PCR. Each bar represents relative expression level of the *fimA*, which was normalized to that of *16 S rRNA* gene. Standard deviations are indicated. Asterisk indicates a statistically significant difference in expression level of *fimA* compared to that in *P*. *gingivalis* without exposure to *S*. *cristatus* (n = 3; *t* test; *p* < 0.05).

To test if *P*. *gingivalis*-*S*. *cristatus* communication occurs through direct cell-cell contact, *P*. *gingivalis* 33277 and *S*. *cristatus* CC5A or its *arcA* mutant were separated using a transwell system with a membrane of pore size either 0.4 µm or 8 µm. After 16 h, bacteria in each the lower well were collected, and numbers of *P*. *gingivalis* 33277 and *S*. *cristatus* CC5A were determined using qPCR. 7.8 × 10^4^ from an input of 1 × 10^7^ CC5A cells migrated to the lower well from the Transwell insert through 8 µm pores, whereas less than 1.5 *S*. *cristatus* cells were detected in the lower well when using the membrane with 0.4 µm pore (Fig. 1a). *P*. *gingivalis* RNA was then purified and expression of the *fimA* gene measured using qRT-PCR. Levels of *fimA* expression were reduced about 2.5 fold when the 8-µm pore transwell was used (Fig. 1b). Inhibition of *fimA* expression by *S*. *cristatus* was not observed when *P*. *gingivalis*-*S*. *cristatus* contact was blocked by the 0.4 µm pore membrane, suggesting that direct contact is required for cell-cell communication between *P*. *gingivalis* and *S*. *cristatus*.

**Figure 2:**
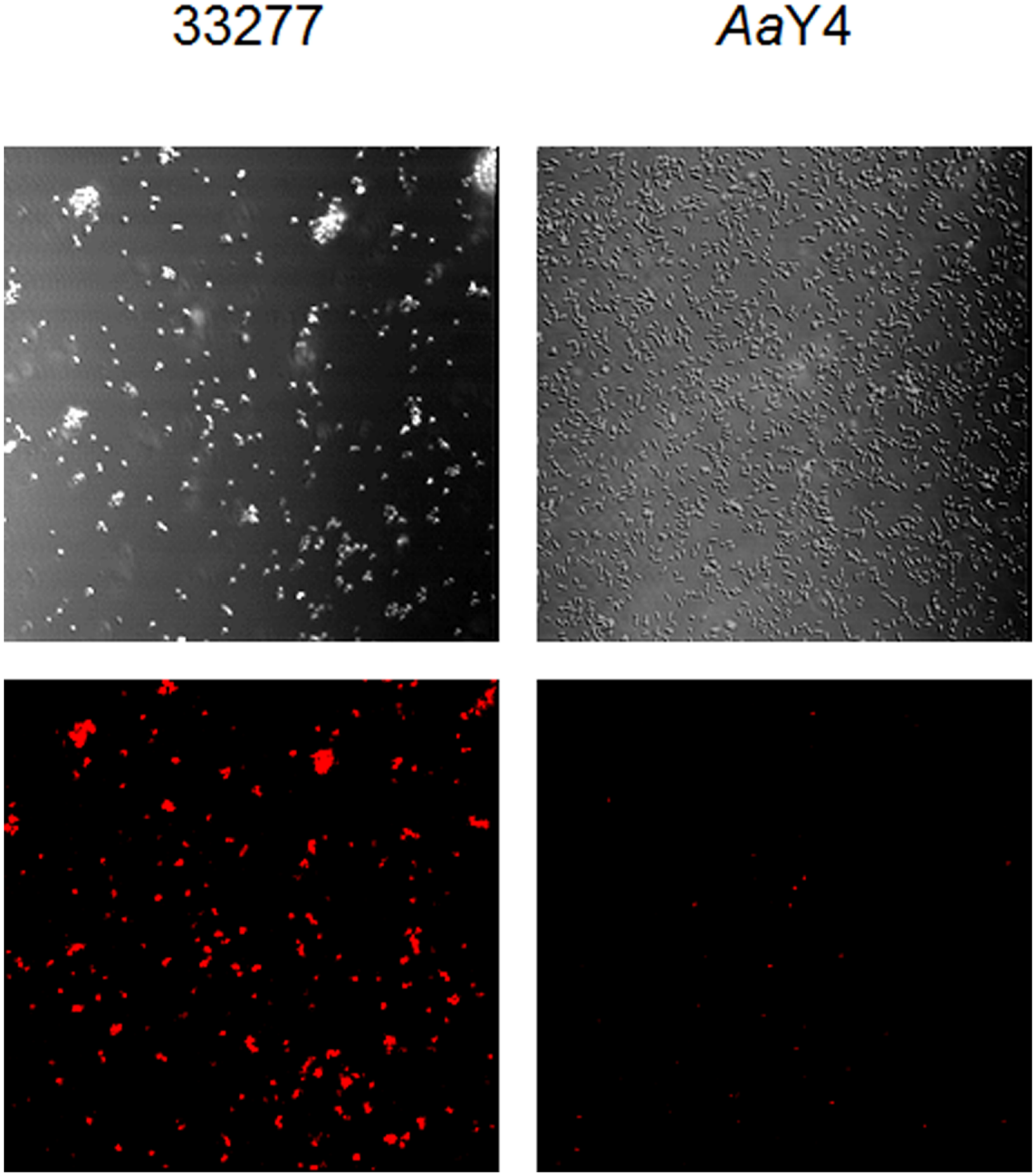
Immunofluorescence antibody images of the interaction of *P*. *gingivalis* 33277 or *A*. *actinomycetemcomitans* Y4 with ArcA. The upper panel presents differential interference contrast (DIC) images showing the location of the bacteria. The lower panels are the TRITC fluorescence labeling (red) images showing bacterial-associated ArcA. Bar is 5 µm.

Direct interaction of *P*. *gingivalis* and *S*. *cristatus* ArcA was confirmed by an immunofluorescence assay with *P*. *gingivalis* cells and purified ArcA protein. Fluorescent labeled *P*. *gingivalis*-ArcA complexes were detected by confocal microscopy. As shown in Fig. 2, ArcA had high affinity for *P*. *gingivalis* 33277, but not for AaY4, suggesting a specific interaction between ArcA and *P*. *gingivalis* surface molecules.

### Isolation of *P*. *gingivalis* surface protein(s) that interacts with ArcA of *S*. *cristatus*

**Figure 3:**
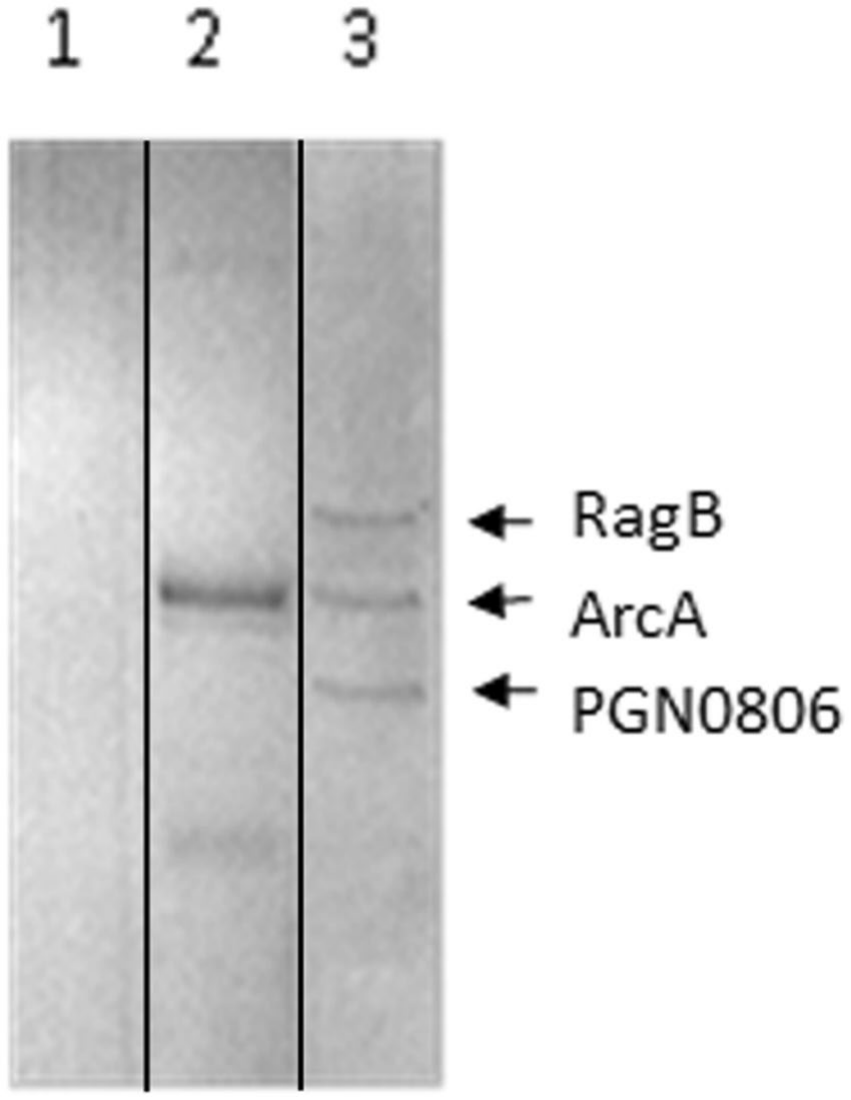
Interaction of ArcA and *P*. *gingivalis* surface proteins. SDS-PAGE analysis of proteins eluted from Sepharose 4B column. Lane 1, the proteins eluted from untreated Sepharose 4B column exposed to *P*. *gingivalis* 33277 extract; Lane 2, the proteins eluted from ArcA antibody-coupled Sepharose 4B column exposed to CC5A extract only; lane 3, the proteins eluted from ArcA antibody-coupled Sepharose 4B column exposed to *P*. *gingivalis* and CC5A extracts. Proteins were stained with Coomassie blue.

To isolate and identify *P*. *gingivalis* surface molecule(s) that interact with ArcA, we performed a pull-down assay. An ArcA antibody coupled Sepharose 4B column was used to capture ArcA-interacting components from a mixture of *P*. *gingivalis* cell lysate and ArcA protein. The proteins eluted from the column were analyzed with SDS-PAGE. Three bands with molecular sizes of approximately 55, 47, and 30 kDa were detected (Fig. 3). Western blot using ArcA antibody showed that the 47 kDa protein is ArcA of *S*. *cristatus* (data not shown). The other two bands were identified by MS analysis as *P*. *gingivalis* RagB (PGN_0294) and a MotA/TolQ/ExbB proton channel family protein (PGN_0806), suggesting that these two proteins are receptors for ArcA.

### Identification of the key functional motif of ArcA

**Figure 4:**
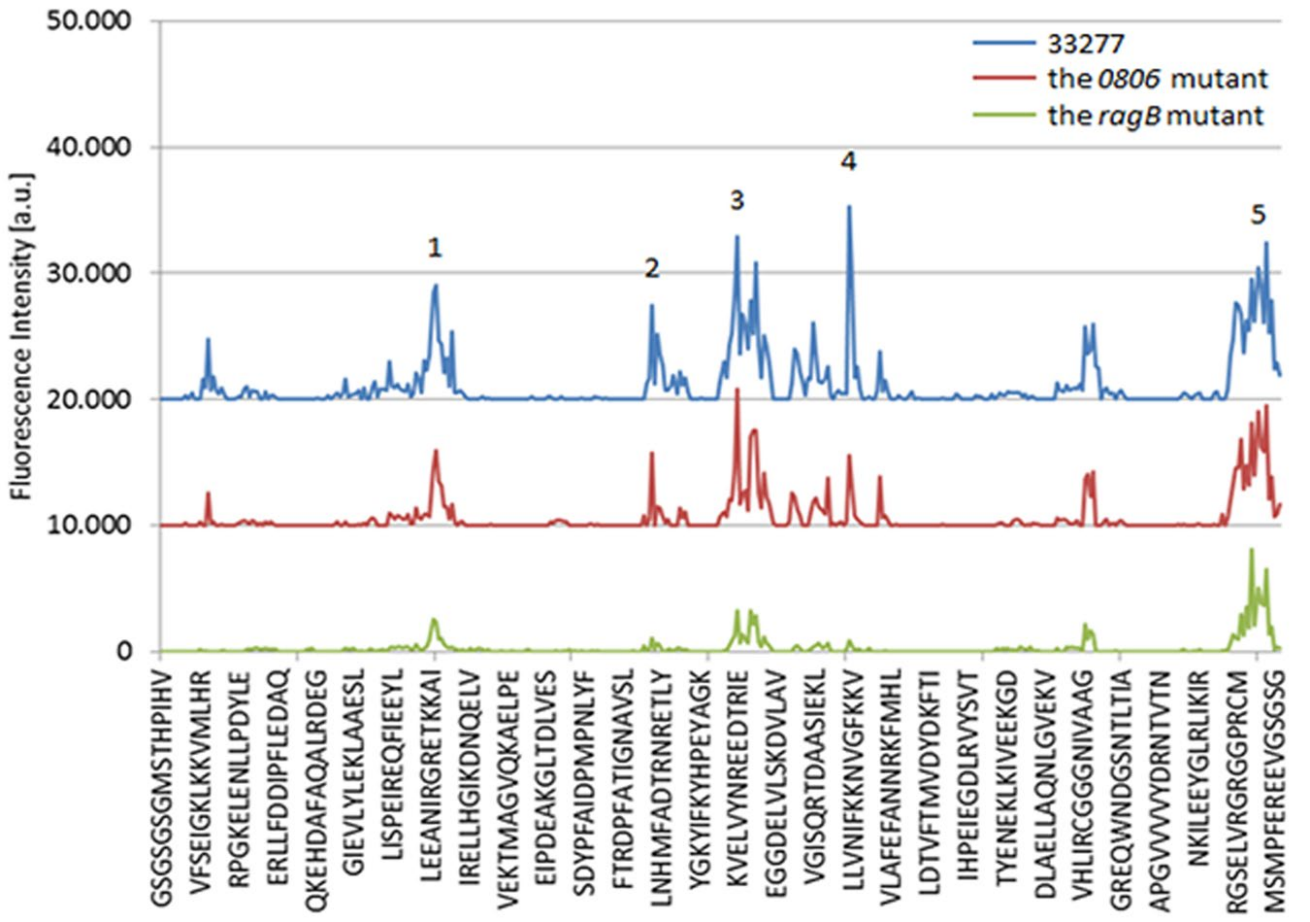
Identification of a binding region of ArcA interacting with *P*. *gingivalis*. A peptide array of ArcA was exposed to *P*. *gingivalis* 33277 and the *ragB* and *pgn0806* mutants. The intensity plot of the peptide array signals shows as peaks with corresponding regions of ArcA. The five of the highest peaks are numbered.

*S*. *cristatus* ArcA is a 47 kDa protein with 409 amino acids^16^. We sought to identify key amino acids and the motif(s) of ArcA responsible for its inhibitory activity toward *fimA* expression. A peptide microarray was first performed to detect binding sites of ArcA for *P*. *gingivalis*. The arrays were incubated with surface extracts of *P*. *gingivalis* 33277, or the Δ*ragB* or *Δ0806* mutants, and binding was detected with *P*. *gingivalis* antibodies. Although the absolute binding capacities (fluorescence intensity) of these strains were significantly varied, likely due to protein degradation of surface extract in some strains, the overall patterns were consistent. Of several peaks observed (Fig. 4), a peptide with the sequence NIFKKNVGFKK (peak 4) and spanning amino acid residues 249–259, was found to have the highest binding affinity to *P*. *gingivalis* 33277 proteins, evident as the highest peak. This peak was no longer the highest when the arrays were incubated with surface extracts isolated from the Δ*0806* or Δ*ragB* mutants, corroborating the involvement of *P*. *gingivalis* proteins PGN_0806 and RagB in recognition of ArcA.

**Figure 5:**
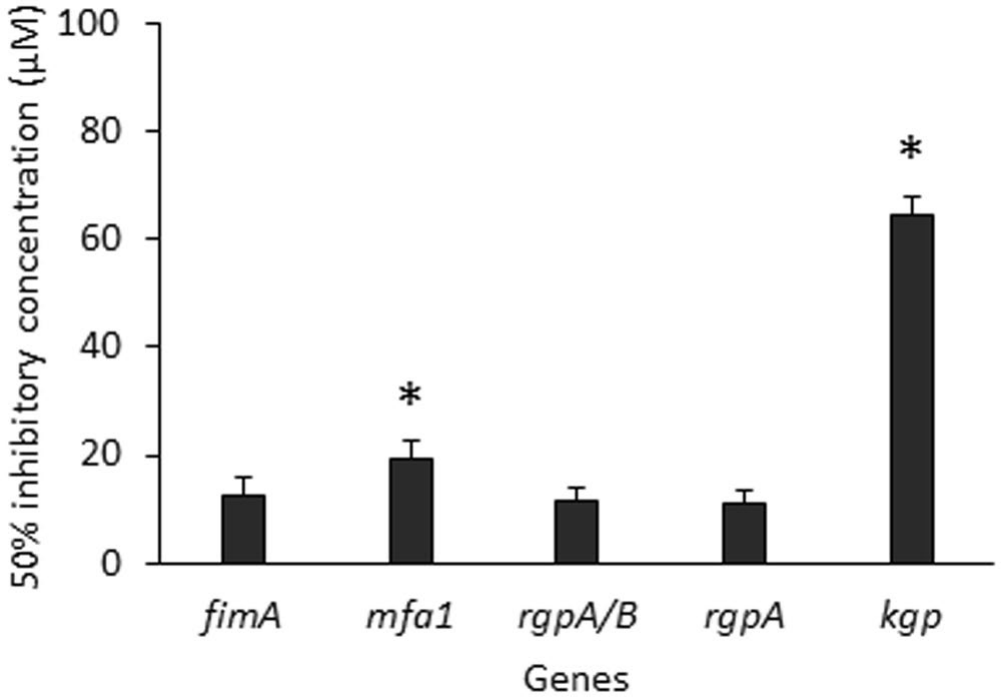
Potency of peptide4 for inhibition of virulence gene expression in *P*. *gingivalis*. The half inhibitory concentration (IC50) was measured by conducting three independent experiments to determine mRNA levels of *fimA*, *mfa1*, *rgpA*/*B*, and *kgp* in the presence of peptide4 at the concentrations 0, 4, 16, and 64 µM, respectively. The IC50 for each gene was established using a Microsoft Excel program with add-in for curve fitting. Asterisks indicate the statistical significances of IC50 of peptide4 for a specific gene when compared to that for the *fimA* gene (*P* < 0.05; *t* test).

Five peptides (1–5 in Table 1) were synthesized based on ArcA array peaks, and the effect of each peptide on gene expression was determined by inclusion of the peptides in the *P*. *gingivalis* growth media. As shown in Table 1, the 11 residue peptide4 from the C-terminal region of ArcA repressed expression of *fimA*, *mfa1*, *kgp*, *rgpA*/*B* (encoding catalytic regions of *rgpA*/*B*), and *rgpA* (encoding adhesin domains of RgpA) genes by at least 2 fold, at a concentration of 16 µM. Expression of *pgn_0128* encoding immunoreactive 53 kDa antigen was not modulated in response to the presence of peptide4, indicating specificity for a subset of virulence-associated genes. Increased inhibitory activity (60–70%) was observed at a concentration of 64 µM (not shown), suggesting that this region is likely a key active motif of ArcA. These results also establish that the effects of ArcA on *P*. *gingivalis* virulence extend beyond repression of *fimA* and include genes for the gingipains and for the minor fimbrial subunit, as also shown by others^17^. The half inhibitory concentration (IC50) was determined by constructing a dose-response curve (0, 4, 16, and 64 µM) to measure the effectiveness of peptide4 in repressing expression of these genes. As shown in Fig. 5, the highest efficiency of peptide4 was found in inhibition of *rgpA* (amplified with primers corresponding to the region encoding the binding domain of RgpA, HGP44), *rgp*A/B (amplified with primers corresponding to the region encoding catalytic regions of RgpA and B), and *fimA* with IC50s of 11.2 ± 2.1, 11.7 ± 2.3 or µM, 12.4 ± 3.4, respectively. Peptide4 also showed a significantly lower IC50 for *mfa1* (19.2 ± 3.3 µM) compared to that of *kgp* (64.3 ± 3.8 µM). It should be pointed out that peptide1, 2, 3 and 5 (Table 1) also exhibited some inhibitory activity, although at a lower efficiency. These regions along with peptide4 may be involved in formation of a structural motif that may have a higher binding capacity than peptide4 alone. These findings provide a molecular basis for the future design of inhibitors of *P*. *gingivalis*.

**Table 1:** Differential expression of virulence genes in *P*. *gingivalis* in the presence of ArcA peptides.

Peptide	Peptide sequence and residue position	Relative expression level^a^					
		*fimA*	*mfa1*	*rgpA*/*B*	*rgpA*	*kgp*	*pgn0128*
P1	I_97_RGRETKK	0.88 ± 0.08	0.85 ± 0.05	0.89 ± 0.05	0.86 ± 0.07	1.06 ± 0.12	0.96 ± 0.06
P2	N_177_HMFADTRNRE	0.80 ± 0.03	0.80 ± 0.10	0.78 ± 0.07	0.81 ± 0.03	0.90 ± 0.05	0.88 ± 0.03
P3	V_208_YNREEDTRIEGGDEL	0.87 ± 0.10	0.82 ± 0.07	0.98 ± 0.06	0.84 ± 0.06	0.99 ± 0.12	0.91 ± 0.07
P4	N_249_IFKKNVGFKK	0.40 ± 0.06*	0.51 ± 0.05*	0.38 ± 0.04*	0.39 ± 0.04*	0.47 ± 0.06*	0.94 ± 0.06
P5	E_389_LVRGRGGPRCMSMPF	0.97 ± 0.05	0.83 ± 0.04	0.72 ± 0.05	0.73 ± 0.06	0.96 ± 0.12	0.91 ± 0.06

^a^ *P*. *gingivalis* 33277 was grown TSB in the presence or absence of peptide at a concentration 16 µM. Transcript levels were measured by real-time PCR. The mRNA levels of genes are indicated relative to the expression level in the absence of peptides as 1 unit. Results shown are means and standard deviations from three independent experiments. Asterisks indicate the statistical significance of expression levels at least two fold in *P*. *gingivalis* grown in TSB with/without peptides (*P* < 0.05; *t* test).

**Figure 6:**
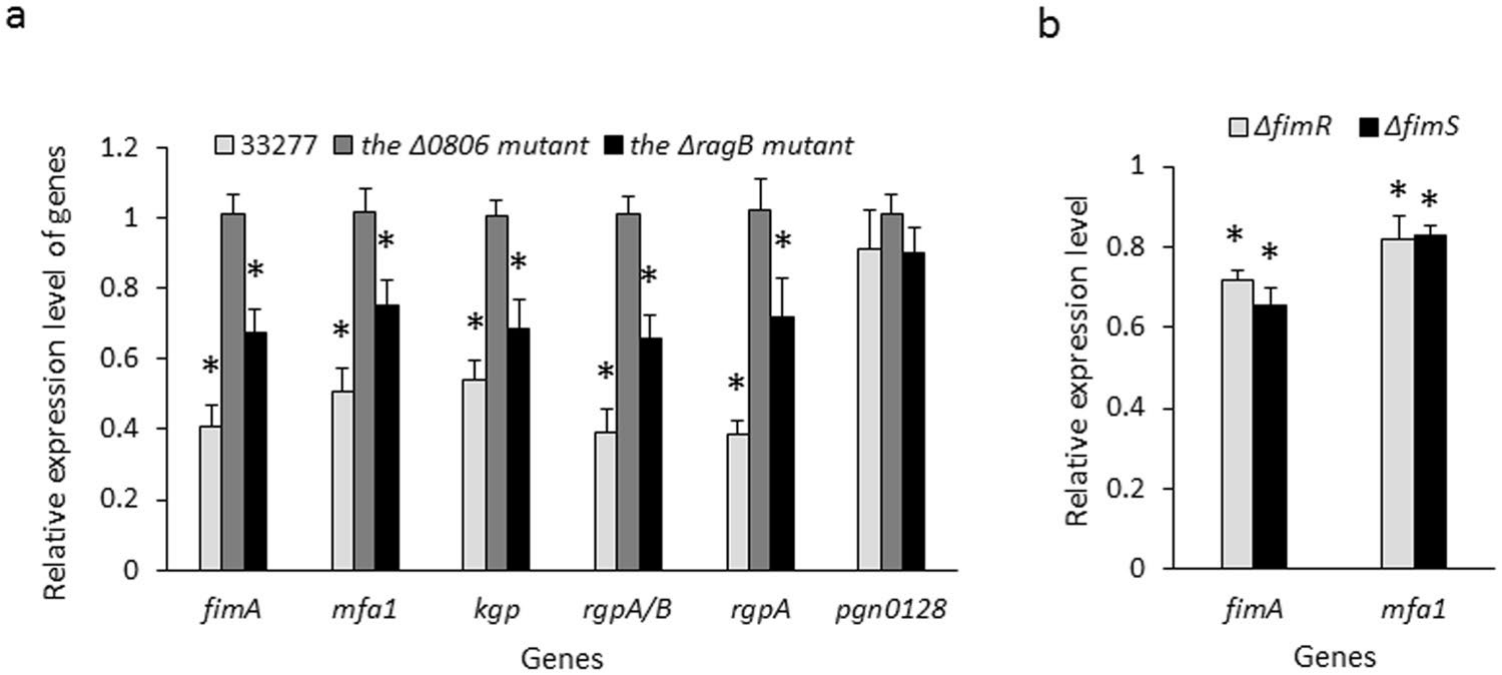
Comparison of virulence gene expression in *P*. *gingivalis* 33277 and its mutants. Expression of *fimA*, *mfa1*, *rgpA* + *B*, *rgpA*, and *kgp* was determined using qRT-PCR. *P*. *gingivalis* strains was grown TSB in the presence or absence of peptide4 at a concentration 16 µM. (**a**) The mRNA levels of genes in 33277, the *pgn_0806*, and the *ragB* mutants grown in the media supplemented with peptide4 are indicated relative to the expression level in *P*. *gingivalis* 33277 grown in the medium without peptide4 (1 unit). (**b**) The *fimR* (ΔfimR) and *fimS* (ΔfimS) mutants were grown with or without the peptide4. Each bar represents relative expression level of *fimA* or *mfa1* in the mutants grown with peptide4 (16 µM) to those in the mutant grown in the media without peptide4 (1 unit). Results shown are means and standard deviations from three independent experiments. Asterisks indicate the statistical significance of expression levels of genes in *P*. *gingivalis* strains grown with/without peptides (*P* < 0.05; *t* test).

To verify that PGN_0806 and RagB function as receptors in *P*. *gingivalis*-*S*. *cristatus* communication, we tested gene expression in the Δ*0806* and Δ*ragB* strains in the presence or absence of peptide4 and compared these to that in the wild type strain 33277. The results showed that loss of PGN_0806 prevented peptide4-dependent regulation of *fimA*, *mfa1*, *rgp*, and *kgp* (Fig. 6a). Although the *ragB* mutation did not completely block peptide4 activity, a significantly reduced inhibitory effect was observed toward all of the target genes. Previously, a two component regulatory system (FimS/R) was identified to be activator of the *fimA* expression^22, 23^. We thus tested the role of FimS/R in *S*. *cristatus*-*P*. *gingivalis* cell-cell communication. Although expression levels of *fimA* and *mfa1* were repressed approximately 20 and 4 fold in the *fimS* and *fimR* mutants (data not shown), Peptide4 mediated regulation of FimA expression reminded intact in the absence of FimS and FimR (Fig. 6b), suggesting FimS/R is not involved in this bacterial cell-cell communication. These results provide strong evidence that PGN_0806 and RagB, either separately or in combination, act as receptors in the bacterial cell-cell communication between *P*. *gingivalis* and *S*. *cristatus*.

**Figure 7:**
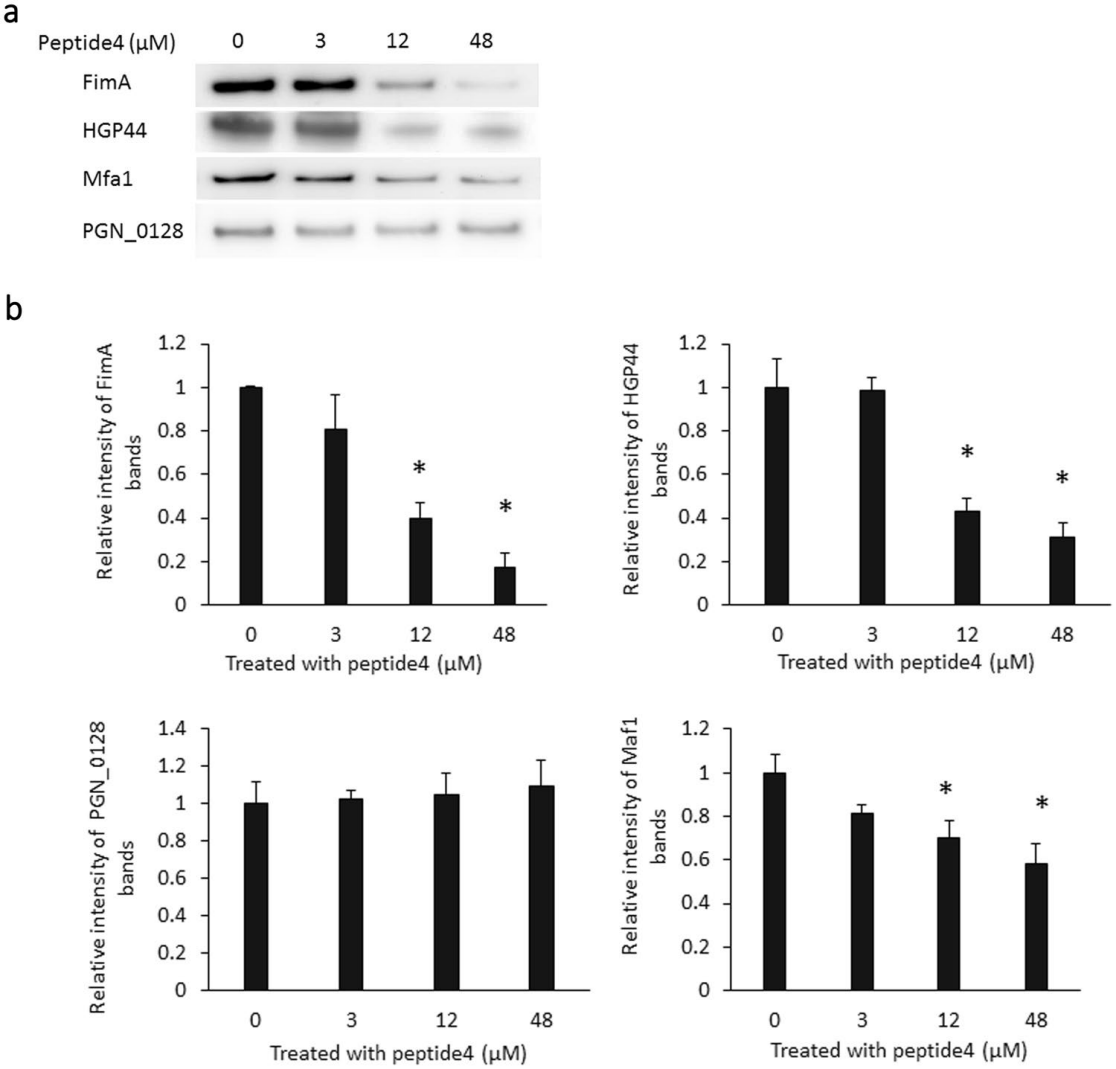
Production of fimbrial proteins and gingipains in *P*. *gingivalis* in response to peptide4. (**a**) Expression levels of FimA, Mfa1, Hgp44 of gingipains, and PGN_0128 (immunoreactive 53 kDa antigen) in surface extracts of *P*. *gingivalis* 33277 exposed to pwptide4 at concentrations 0, 3, 12, and 48 µM were analyzed using a Western blot analysis. (**b**) Semiquantitation of western blots was conducted with ImageJ software. Each bar represents the intensity of the protein band. An asterisk indicates a significant difference between the relative intensity of the protein bands in *P*. *gingivalis* exposed to peptide4 (3, 12, or 48 µM) compared to those seen in *P*. *gingivalis* not exposed (0 µM, 1 unit) (*P* < 0.05 by *t* test).

**Figure 8:**
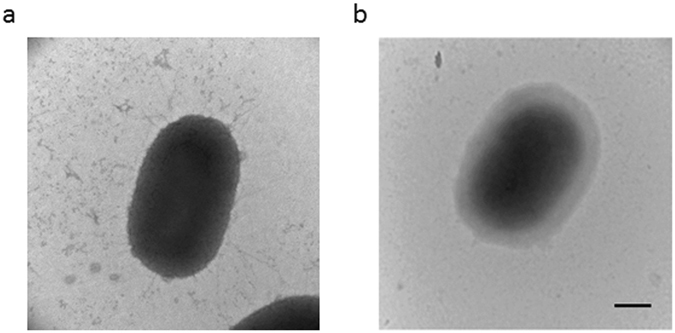
Transmission electron microscopic analysis of *P*. *gingivalis* fimbriae. Fimbrial structures were visualized using TEM. *P*. *gingivalis* strains 33277 (**a**) and 33277 treated with peptide4 (**b**) were grown on the TSB plate for 48 h and prepared by negatively staining with ammonium molybdate. Bars = 0.2 µm.

Expression of fimbrial proteins and gingipains at the translational level was also determined using Western blot analysis. *P*. *gingivalis* 33277 was grown with peptide4 at concentrations of 0, 3, 12, and 48 µM (0×, ¼×, 1×, and 4× IC50 of *fimA* expression) for 48 h. As shown in Fig. 7a,b, production of FimA, Mfa1, and HGP44 (a binding domain of RgpA) was significantly decreased in the presence of 12 and 48 µM of peptide4. However, production of immunoreactive 53 kDa antigen was not altered, consistent with the expression pattern observed at the transcriptional level. Transmission electron microscopy further showed that there were few fimbriae on the surface of *P*. *gingivalis* grown in media supplemented with peptide4 (16 µM), when compared to *P*. *gingivalis* cells grown without peptide4 (Fig. 8a,b).

## Discussion

*P*. *gingivalis* possesses a vast array of effector molecules and systems that enable the organism to colonize and survive in the oral cavity, communicate with other bacteria, and ultimately elevate the virulence of the entire microbial community^24–27^. Major fimbriae (long fimbriae) composed of FimA, are promiscuous adhesins and contribute to colonization, biofilm formation, cell invasion, bone resorption, and the evasion of host defense systems^25, 28–37^. With regard to induction of immune dysbiosis, FimA binds the CXC-chemokine receptor 4 (CXCR4) and induces crosstalk with TLR2 that inhibits the MyD88-dependent antimicrobial pathway^24^. Both the major and minor (Mfa1) fimbriae of *P*. *gingivalis* mediate coadhesion with *S*. *gordonii* and are thus involved in synergistic pathogenicity^38–40^. The majority of *P*. *gingivalis* clinical isolates are fimbriated, especially those isolated at the base of periodontal pockets^41–43^. Other well-known virulence factors are the gingipains which include two arginine- and one lysine-specific cysteine proteinases (RgpA, RgpB, and Kgp)^44^. Thus far, all tested *P*. *gingivalis* strains produce gingipains that are both membrane-associated and secreted soluble forms^45^. Besides their role in tissue matrix destruction due to proteolytic activity^46^, gingipains play an important role in biofilm formation of *P*. *gingivalis* through the C-terminal adhesive regions of RgpA and Kgp or through processing profimbrillin^47, 48^. Gingipains are also involved in modulating immune responses, by cleavage of secreted chemokines and intracellular immune kinases^49, 50^. Previously, we reported that *S*. *cristatus* ArcA represses *fimA* expression in *P*. *gingivalis* ^16, 51^. Similar results, reported by others^17, 52^, showed downregulation of both *fimA* and *mfa1* fimbriae by *Streptococcus intermedius* ArcA. In these studies ArcA enzymatic activity is required for an effect of on biofilm formation through arginine depletion, suggesting an additional indirect role of ArcA in *P*. *gingivalis* colonization^52^. These observations suggest that ArcA modulates expression of fimbrial proteins in *P*. *gingivalis* both directly and indirectly. Collectively, accumulating observations suggest that ArcA modulates expression of fimbrial proteins in *P*. *gingivalis* both directly and indirectly. Here, we identified a functional motif of ArcA, located at the C-terminal and spanning amino acids 249–259, and a peptide (peptide4) derived from this region showed inhibitory activity for both mRNA and protein expression of fimbriae (FimA and Mfa1) and gingipains (RgpA/B and Kgp). Hence this peptide is a potential candidate for developing inhibitors against *P*. *gingivalis*. Based on our observation that ArcA specifically binds to the surface of *P*. *gingivalis*, it is likely that the peptide inhibitors would be specific for this organism and not have a significant inhibitory effect on early biofilm colonizers (streptococci and actinomyces). Targeting *P*. *gingivalis* alone would likely be sufficient to impede the development of a dysbiotic biofilm, as *P*. *gingivalis* is considered a keystone pathogen^8, 53^.

Cell surface receptors are important elements in signal transduction, and possess the ability to bind (sense) a specific signal, subsequently eliciting a specific cellular response. A well-known signal transduction process in bacteria involves two-component regulatory systems which involve a sensor histidine kinase and a response regulator protein^54^. The FimS/R two-component signal transduction in *P*. *gingivalis* predominantly regulates *fimA* expression^55, 56^ and some other genes including *mfa1* ^22, 23^. However, results from the current study showed that FimS/R was not involved in communication between *P*. *gingivalis* and *S*. *cristatus*, since expression levels of *fimA* and *mfa1* in the *fimS* or *fimR* mutants were also modulated in response to peptide4. However, we identified two *P*. *gingivalis* surface proteins, RagB (PGN_0294), a major immunodominant antigen of *P*. *gingivalis*, and PGN_0806 annotated as a MotA/TolQ/ExbB proton channel family protein, which interact with ArcA of *S*. *cristatus*, especially with the peptide4 region of ArcA. Interestingly, the *Pseudomonas aeruginosa* TonB–ExbB–ExbD protein complex is reported to be involved in signal transduction^57^. Moreover, RagA, which is thought to associate with RagB on the *P*. *gingivalis* surface, is a TonB-dependent receptor^58–60^. We also demonstrate that mutation in the *ragB* gene partially blocks the inhibitory activity of ArcA against *fimA*, while the *P*. *gingivalis* strain carrying mutation in the *pgn_0806* gene was completely abrogated in response to peptide4. These data corroborate the role of PGN_0806 and RagB, as receptors, in *P*. *gingivalis-S*. *cristatus* communication. Although we identified a signal peptide and potential receptor(s), the mechanisms of intracellular signal transduction are still unidentified. A previous study showed that expression of *rgpA*, but not *kgp*, was decreased in a *prtT* mutant, which indicated that expression of *kgp* and *rgp* is not coordinately regulated^61^. Therefore, we speculate that independent intracellular transmitters are involved in control of *fimA*, *mfa1*, *kgp*, and *rgp*.

In conclusion, we have identified a functional domain of *S*. *cristatus* ArcA that has high affinity to the surface of *P*. *gingivalis* and is able to repress expression of several well-known virulence genes involved in production of fimbriae and gingipains. We also uncovered two surface proteins of *P*. *gingivalis*, RagB and PGN_0806, which interact with ArcA and are required for bacterial cell-cell communication between *P*. *gingivalis* and *S*. *cristatus*. These results functionally characterize and molecularly dissect the antagonistic relationship between these oral bacteria that we reported earlier^16, 62^. Application of these findings should provide the basis for therapeutic strategies designed to reduce colonization of *P*. *gingivalis* in the oral cavity and suppress the pathogenicity of periodontitis-associated dental plaque.

## Methods

### Bacterial strains and growth conditions

*P*. *gingivalis* strains and *A*. *actinomycetemcomitans* Y4 were grown from frozen stocks in Trypticase soy broth (TSB) or on TSB blood agar plates supplemented with yeast extract (1 mg/ml), hemin (5 μg/ml), and menadione (1 μg/ml), and incubated at 37 °C in an anaerobic chamber (85% N_2_, 10% H_2_, 5% CO_2_). *S*. *cristatus* CC5A and isogenic ΔarcA^16^ were grown in Trypticase peptone broth (TPB) supplemented with 0.5% glucose at 37 °C under aerobic conditions. Erythromycin (5 μg/ml) or tetracycline (0.5 μg/ml) were added to growth media when appropriate.

### Transwell co-culture assay

*P*. *gingivalis* cells (10^5^) were inoculated in each well of a six well plate, and *S*. *cristatus* CC5A or its *arcA* mutant (10^7^) was added into the transwell inserts with a polycarbonate porous membrane of pore size 0.4 µm or 8 µm. After 16 h growth, the bacterial cells in each well of the plate were collected by centrifugation. To determine the number of CC5A migrated to the lower wells, the bacteria in the lower wells were harvested by centrifugation, and DNA was released by boiling the samples for 20 mins. Numbers of bacteria were determined by qPCR using specific primers for CC5A *arcA* and 33277 *16s-rRNA* (Table S1). *P*. *gingivalis* RNA was purified using an RNeasy mini spin column which selectively lyses *P*. *gingivalis* cells and not *S*. *cristatus* cells. Expression of the *fimA* gene in *P*. *gingivalis* was measured using real time qRT-PCR (see below).

### RNA isolation and qPCR

*P*. *gingivalis* were homogenized in Trizol Reagent (Invitrogen, Carlsbad, CA) and RNA was purified using an RNeasy mini spin column (Qiagen, Valencia, CA). RNA samples were digested on-column with RNase-free DNase, and total RNA was tested using an Agilent 2100 Bioanalyzer to ensure the quality of the samples. Primers are listed in Table S1. Amplification reactions consisted of a reverse transcription using a Bio-Rad iScript Reverse Transcription Supermix on a TC-3000 thermal cycler (Techne, Staffordshire, ST15 OSA, UK) and a real-time qRT-PCR analysis using a QuantiTect SYBR Green RT-PCR Kit (Qiagen) on an iCycler MyiQ^TM^ Real-Time PCR detection system (Bio-Rad Laboratories, Inc, Hercules, CA) according to the manufacturer’s instructions. *P*. *gingivalis 16 S rRNA* gene was used as a normalizing gene. The melting curve profile was analyzed to verify a single peak for each sample, which indicated primer specificity. The expression levels of the investigated genes for the test sample were determined relative to the untreated calibrator sample by using the comparative cycle threshold (*ΔC*_*T*_) method. *ΔC*_*T*_ was calculated by subtracting the average *C*_*T*_ value of the test sample from the average *C*_*T*_ value of the calibrator sample, and the value used to calculate the ratio between the two by assuming 100% amplification efficiency. By loading the same amount of total RNA for any comparable samples, the *ΔC*_*T*_ represents the difference in gene expression between the samples.

### Confocal microscopy

ArcA (50 µg) was purified from *S*. *cristatus* CC5A as described^22^, mixed with *P*. *gingivalis* cells (10^8^) in PBS and incubated at room temperature for 1 h. After washing three times with PBS, bacteria-protein complexes were blocked in PBS with 5% BSA for 1 h. ArcA bound to *P*. *gingivalis* was detected by staining with rabbit anti-ArcA polyclonal antibody (1:400) and tetramethyl rhodamine isothiocyanate (TRITC, 1:500)-conjugated AffiniPure Goat Anti-Rabbit IgG antibody. Visualization was with a LSM 510 inverted confocal microscope with selected filters (543 nm excitation and 560 nm emission).

### Pull-down assays

To isolate and identify *P*. *gingivalis* surface molecule(s) that interacts with ArcA, we performed a pull-down assay. Surface extracts of *P*. *gingivalis* were prepared by sonication with a Sonic Dismembrator (Fisher Scientific; output control 8, 20 s × 3), and the cell debris were removed by centrifugation followed by filtration (0.2-μm pore size). Surface extracts of 33277 were mixed with purified ArcA on a rotator at room temperature for 1 h, and then added to an ArcA antibody coupled Sepharose 4B column (Sigma-Aldrich). After incubation at room temperature for 1 h, the column was washed three times with PBS containing 0.01% Tween, and proteins were eluted by 0.1 M Glycine pH 2.4. After SDS-PAGE, bands were excised and identified by liquid chromatography–mass spectrometry.

### Construction of the *pgn_0294* (*ragB)* and *pgn_0806* (*motA*/*tolQ*/*exbB*) mutants

Insertional mutants (*pgn_0294* and *pgn_0806*) were generated by ligation-independent cloning of PCR mediated mutagenesis (LIC-PCR)^63–65^. A 2.1-kb *ermF-ermAM* cassette was introduced into target genes by three step PCR to yield *pgn_0294-erm- pgn_0294* or *pgn_0806-erm-pgn_0806* DNA fragments as described previously^64^. The final PCR products were then introduced into *P*. *gingivalis* 33277 by electroporation. Mutants were selected on TSB plates containing erythromycin (5 µg/ml). The insertional mutation was confirmed by PCR analysis, and the mutants were designated as *P*. *gingivalis* Δ0806 or ΔragB.

### Protein Interaction Screening

Surface extracts of *P*. *gingivalis* were collected by sonication (1 min for three times) and centrifugation (13 000 g for 30 min) followed by filtration (0.2 μm pore size). Peptide microarray analysis was performed by PEPperPRINT (Heidelberg, Germany). An ArcA microarray was generated with 409 different peptides of ArcA, and each peptide contained 15 amino acids with a peptide-peptide overlap of 14 amino acids. After blocking and washing, the array was incubated with surface extracts (100 µg/ml) isolated from *P*. *gingivalis* 33277, the *0806* mutant, or the *ragB* mutant. *P*. *gingivalis* surface proteins bound on the ArcA array were detected using anti-*P*. *gingivalis* antibodies and sheep anti-rabbit IgG (H + L) DyLight680. The arrays were analyzed with a LI-COR Odyssey Imaging System.

### Peptide synthesis and activity

Peptides were synthesized by PEPperPRINT and Biomatik (Delaware) and purified with High Performance Liquid Chromatography (HPLC) at ≥90% purity. Peptides were resuspended in nuclease/proteinase-free PBS, aliquoted, and stored at −20 °C. Inhibitory activity of peptides were determined as described^16^ with slight modification. Peptides were mixed with 5 × 10^5^ cells of *P*. *gingivalis*, spotted onto a TSB blood agar plate, and cultured for 60 h anaerobically. The expression levels of fimbrial and gingipain mRNA or proteins were measured using qRT-PCR or western blot analyses.

### Western Blot Analysis

*P*. *gingivalis* cells were lysed using BugBuster® Protein Extraction Reagent (EMD Millipore, Darmstadt, Germany), and the protein concentration determined using a Bio-Rad protein assay (Bio-Rad). Lysates (0.5 µg) were separated by 12% sodium SDS-PAGE, and transferred to nitrocellulose membranes (Invitrogen, Carlsbad, CA) with a Mini Transblot Electrophoretic transfer cell (Bio-Rad) at 100 V for 1 h. The membrane was blocked with 3% BSA in PBS for 1 h and incubated with polyclonal anti-FimA, anti-Mfa1, anti-HGP44 (a C-terminal adhesin domain of gingipains), or anti-PGN-0128 antibodies diluted 1:1,000 for 1 h. After washing with PBS, the membrane was incubated with anti-rabbit horseradish peroxidase-conjugated secondary antibodies for 1 h. The proteins were visualized using enhanced chemiluminescence (GE Healthcare Bio-Sciences Corp, Pittsburgh, PA) and measured using a semi-quantitative Western blot technique with ImageJ software (NIH, Bethesda, Maryland).

### Transmission electron microscopy

*P*. *gingivalis* cells were grown on TSB blood plates for 48 h with or without peptide4. Bacterial cells were collected and resuspended in PBS. 20 µl of bacterial suspension were applied to a Formvar-coated copper grid (200 mesh, Electron Microscopy Sciences, PA) and air dried. The bacterial cells were then negatively stained with 0.5% ammonium molybdate for 4 min and observed with a transmission electron microscope (Philips CM-12, Portland, OR) operated at 80 kV.

### Statistical analyses

A student’s *t*-test was used to determine the statistical significance of differences in gene expression profiles and growth rates of *P*. *gingivalis* strains. A *p* < 0.05 was considered significant.

## Electronic supplementary material

Oligonucleotide primers used in this study

## References

[1] Kassebaum NJ (2014). Global burden of severe periodontitis in 1990–2010: a systematic review and meta-regression. Journal of dental research.

[2] Burt B, Research S (2005). & Therapy Committee of the American Academy of, P. Position paper: epidemiology of periodontal diseases. Journal of periodontology.

[3] Petersen PE, Ogawa H (2012). The global burden of periodontal disease: towards integration with chronic disease prevention and control. Periodontology.

[4] Eke PI (2015). Update on Prevalence of Periodontitis in Adults in the United States: NHANES 2009 to 2012. Journal of periodontology.

[5] Hajishengallis G (2015). Periodontitis: from microbial immune subversion to systemic inflammation. Nature reviews. Immunology.

[6] Linden GJ, Lyons A, Scannapieco FA (2013). Periodontal systemic associations: review of the evidence. Journal of periodontology.

[7] Kumar PS (2013). Oral microbiota and systemic disease. Anaerobe.

[8] Hajishengallis G, Lamont RJ (2012). Beyond the red complex and into more complexity: the polymicrobial synergy and dysbiosis (PSD) model of periodontal disease etiology. Molecular oral microbiology.

[9] Hajishengallis G (2011). Low-abundance biofilm species orchestrates inflammatory periodontal disease through the commensal microbiota and complement. Cell host & microbe.

[10] Hajishengallis G, Lamont RJ (2014). Breaking bad: manipulation of the host response by Porphyromonas gingivalis. European journal of immunology.

[11] Hajishengallis G, Darveau RP, Curtis MA (2012). The keystone-pathogen hypothesis. Nature reviews. Microbiology.

[12] Darveau RP, Hajishengallis G, Curtis MA (2012). Porphyromonas gingivalis as a potential community activist for disease. Journal of dental research.

[13] Page RC (2007). Immunization of Macaca fascicularis against experimental periodontitis using a vaccine containing cysteine proteases purified from Porphyromonas gingivalis. Oral microbiology and immunology.

[14] Daep CA, Novak EA, Lamont RJ, Demuth DR (2011). Structural dissection and *in vivo* effectiveness of a peptide inhibitor of Porphyromonas gingivalis adherence to Streptococcus gordonii. Infection and immunity.

[15] Xie H, Hong J, Sharma A, Wang BY (2012). Streptococcus cristatus ArcA interferes with Porphyromonas gingivalis pathogenicity in mice. Journal of periodontal research.

[16] Xie H, Lin X, Wang BY, Wu J, Lamont RJ (2007). Identification of a signalling molecule involved in bacterial intergeneric communication. Microbiology.

[17] Christopher AB, Arndt A, Cugini C, Davey ME (2010). A streptococcal effector protein that inhibits Porphyromonas gingivalis biofilm development. Microbiology.

[18] Burne RA, Marquis RE (2000). Alkali production by oral bacteria and protection against dental caries. FEMS microbiology letters.

[19] Curran TM, Lieou J, Marquis RE (1995). Arginine deiminase system and acid adaptation of oral streptococci. Appl Environ Microbiol.

[20] Lin X, Lamont RJ, Wu J, Xie H (2008). Role of differential expression of streptococcal arginine deiminase in inhibition of fimA expression in Porphyromonas gingivalis. Journal of bacteriology.

[21] Wang BY, Wu J, Lamont RJ, Lin X, Xie H (2009). Negative correlation of distributions of Streptococcus cristatus and Porphyromonas gingivalis in subgingival plaque. Journal of clinical microbiology.

[22] Wu J, Lin X, Xie H (2007). Porphyromonas gingivalis short fimbriae are regulated by a FimS/FimR two-component system. FEMS microbiology letters.

[23] Lo A (2010). FimR and FimS: biofilm formation and gene expression in Porphyromonas gingivalis. Journal of bacteriology.

[24] Hajishengallis G, McIntosh ML, Nishiyama SI, Yoshimura F (2013). Mechanism and implications of CXCR4-mediated integrin activation by Porphyromonas gingivalis. Molecular oral microbiology.

[25] Lamont RJ, Jenkinson HF (1998). Life below the gum line: pathogenic mechanisms of Porphyromonas gingivalis. Microbiology and molecular biology reviews: MMBR.

[26] Lamont RJ, Jenkinson HF (2000). Subgingival colonization by Porphyromonas gingivalis. Oral microbiology and immunology.

[27] Xie H (2015). Biogenesis and function of Porphyromonas gingivalis outer membrane vesicles. Future microbiology.

[28] Goulbourne PA, Ellen RP (1991). Evidence that Porphyromonas (Bacteroides) gingivalis fimbriae function in adhesion to Actinomyces viscosus. J Bacteriol.

[29] Lamont RJ, Bevan CA, Gil S, Persson RE, Rosan B (1993). Involvement of Porphyromonas gingivalis fimbriae in adherence to Streptococcus gordonii. Oral Microbiol Immunol.

[30] Amano A (1997). Prophyromonas gingivalis fimbriae mediate coaggregation with Streptococcus oralis through specific domains. J Dent Res.

[31] Lin X, Wu J, Xie H (2006). Porphyromonas gingivalis minor fimbriae are required for cell-cell interactions. Infection and immunity.

[32] Weinberg A, Belton CM, Park Y, Lamont RJ (1997). Role of fimbriae in Porphyromonas gingivalis invasion of gingival epithelial cells. Infect Immun.

[33] Hajishengallis G, Shakhatreh MA, Wang M, Liang S (2007). Complement receptor 3 blockade promotes IL-12-Mediated Clearance of Porphyromonas gingivalis and Negates Its Virulence *In Vivo*. J Immunol.

[34] Wang M (2007). Fimbrial proteins of porphyromonas gingivalis mediate *in vivo* virulence and exploit TLR2 and complement receptor 3 to persist in macrophages. Journal of immunology.

[35] Malek R (1994). Inactivation of the Porphyromonas gingivalis fimA gene blocks periodontal damage in gnotobiotic rats. J Bacteriol.

[36] Kuboniwa M (2009). Distinct roles of long/short fimbriae and gingipains in homotypic biofilm development by Porphyromonas gingivalis. BMC microbiology.

[37] Enersen M, Nakano K, Amano A (2013). Porphyromonas gingivalis fimbriae. Journal of oral microbiology.

[38] Maeda K (2004). Oral streptococcal glyceraldehyde-3-phosphate dehydrogenase mediates interaction with Porphyromonas gingivalis fimbriae. Microbes Infect.

[39] Park Y (2005). Short fimbriae of Porphyromonas gingivalis and their role in coadhesion with Streptococcus gordonii. Infect Immun.

[40] Daep CA, Lamont RJ, Demuth DR (2008). Interaction of Porphyromonas gingivalis with oral streptococci requires a motif that resembles the eukaryotic nuclear receptor box protein-protein interaction domain. Infect Immun.

[41] Suzuki Y, Yoshimura F, Takahashi K, Tani H, Suzuki T (1988). Detection of fimbriae and fimbrial antigens on the oral anaerobe Bacteroides gingivalis by negative staining and serological methods. J Gen Microbiol.

[42] Suzuki Y, Yoshimura F, Tani H, Suzuki T (1988). Fimbriae from the oral anaerobe Bacteroides gingivalis: a screening of clinical isolates from various places. Adv Dent Res.

[43] Noiri Y, Li L, Yoshimura F, Ebisu S (2004). Localization of Porphyromonas gingivalis-carrying fimbriae *in situ* in human periodontal pockets. J Dent Res.

[44] Curtis MA (1999). Molecular genetics and nomenclature of proteases of Porphyromonas gingivalis. Journal of periodontal research.

[45] Mikolajczyk-Pawlinska J (1998). Genetic variation of Porphyromonas gingivalis genes encoding gingipains, cysteine proteinases with arginine or lysine specificity. Biological chemistry.

[46] Potempa J, Banbula A, Travis J (2000). Role of bacterial proteinases in matrix destruction and modulation of host responses. Periodontology.

[47] Kadowaki T (1998). Arg-gingipain acts as a major processing enzyme for various cell surface proteins in Porphyromonas gingivalis. The Journal of biological chemistry.

[48] Shi Y (1999). Genetic analyses of proteolysis, hemoglobin binding, and hemagglutination of Porphyromonas gingivalis. Construction of mutants with a combination of rgpA, rgpB, kgp, and hagA. The Journal of biological chemistry.

[49] Klarstrom Engstrom K, Khalaf H, Kalvegren H, Bengtsson T (2015). The role of Porphyromonas gingivalis gingipains in platelet activation and innate immune modulation. Molecular oral microbiology.

[50] Barth K, Genco CA (2016). Microbial Degradation of Cellular Kinases Impairs Innate Immune Signaling and Paracrine TNFalpha Responses. Scientific reports.

[51] Wu J, Xie H (2010). Role of arginine deiminase of Streptococcus cristatus in Porphyromonas gingivalis colonization. Antimicrobial agents and chemotherapy.

[52] Cugini C, Stephens DN, Nguyen D, Kantarci A, Davey ME (2013). Arginine deiminase inhibits Porphyromonas gingivalis surface attachment. Microbiology.

[53] Hajishengallis G, Lamont RJ (2016). Dancing with the Stars: How Choreographed Bacterial Interactions Dictate Nososymbiocity and Give Rise to Keystone Pathogens, Accessory Pathogens, and Pathobionts. Trends in microbiology.

[54] Zschiedrich CP, Keidel V, Szurmant H (2016). Molecular Mechanisms of Two-Component Signal Transduction. Journal of molecular biology.

[55] Nishikawa K, Yoshimura F, Duncan MJ (2004). A regulation cascade controls expression of Porphyromonas gingivalis fimbriae via the FimR response regulator. Molecular microbiology.

[56] Hayashi J, Nishikawa K, Hirano R, Noguchi T, Yoshimura F (2000). Identification of a two-component signal transduction system involved in fimbriation of Porphyromonas gingivalis. Microbiology and immunology.

[57] Wirth C, Meyer-Klaucke W, Pattus F, Cobessi D (2007). From the periplasmic signaling domain to the extracellular face of an outer membrane signal transducer of Pseudomonas aeruginosa: crystal structure of the ferric pyoverdine outer membrane receptor. J Mol Biol.

[58] Goulas T (2015). Structure of RagB, a major immunodominant outer-membrane surface receptor antigen of Porphyromonas gingivalis. Molecular oral microbiology.

[59] Hutcherson JA (2015). Porphyromonas gingivalis RagB is a proinflammatory signal transducer and activator of transcription 4 agonist. Molecular oral microbiology.

[60] Nagano K (2007). Characterization of RagA and RagB in Porphyromonas gingivalis: study using gene-deletion mutants. Journal of medical microbiology.

[61] Tokuda M, Chen W, Karunakaran T, Kuramitsu HK (1998). Regulation of protease expression in Porphyromonas gingivalis. Infection and immunity.

[62] Xie H (2000). Intergeneric communication in dental plaque biofilms. Journal of bacteriology.

[63] Wu J, Lin X, Xie H (2008). OxyR is involved in coordinate regulation of expression of fimA and sod genes in Porphyromonas gingivalis. FEMS microbiology letters.

[64] Wu J, Lin X, Xie H (2009). Regulation of hemin binding proteins by a novel transcriptional activator in Porphyromonas gingivalis. Journal of bacteriology.

[65] Aslanidis C, de Jong PJ (1990). Ligation-independent cloning of PCR products (LIC-PCR). Nucleic acids research.

