# Supplementary material for: Identification of Streptococcus cristatus peptides that repress expression of virulence genes in Porphyromonas gingivalis
Source: Sci Rep. 2017 May 3;7:1413. doi: 10.1038/s41598-017-01551-4 (PMC5431200; doi:10.1038/s41598-017-01551-4)
Supplement: Supplementary file 1 — Oligonucleotide primers used in this study [file 41598_2017_1551_MOESM1_ESM.doc]

**Identification of *Streptococcus cristatus* peptides that repress expression of virulence genes in *Porphyromonas gingivalis***

Meng-Hsuan Ho^1^, Richard J. Lamont^2^ and Hua Xie^1^*

**Table S1. Oligonucleotide primers used in this study**

| Gene | Primer name | Primer sequences (5’-3’) |
| --- | --- | --- |
| *fimA* | FimA-88F  FimA-88R | cggaacgaataacccagaga  ctgaccaacgagaacccact |
| *mfa1* | Mfa1-F  Mfa1-R | CAGATGGGTTGTTGCTCA  ATGGAAAGTGCTGCTGGTAG |
| *kgp* | Kgp-133F  Kgp-133R | CTATTGGGAACTGCTGTGTTAC  TCCTCGCCCCAATAAGAATTC |
| *rgp* | Rgp-194F  Rgp-194R | CAACAGCAACCAGCTACCGT  CGTTCATCTCATCCTGCCCG |
| *rgpA* | RgpA-156F  RgpA-156R | GACGGTTATTAAGACCATCAACAC  TCCACGCTGCGAGCGGTAT |
| *sod* | Sod-235F  Sod-235R | AATTCCACCACGGTAAGCAC  GAGCCGAATTGTTTGTCGAT |
| *pgn0128* | Pgn0128-114F  Pgn0128-114R | TAATGGGAAGAGCGAGCAGT  ACAGGGCATTTAGCACAACC |
| *16s-rRNA* | 16s-rRNAF  16s-rRNAR | TGGGTTTAAAGGGTGCGTAG  CAATCGGAGTTCCTCGTGAT |
| *arcA* | arcA186-F  arcA186-R | TCCAATGCCAAACCTTTACT  ATACGAGTATCTTCTTCACG |
